# Radioprotective and Radiomitigative Effects of Melatonin in Tissues with Different Proliferative Activity

**DOI:** 10.3390/antiox10121885

**Published:** 2021-11-25

**Authors:** Serazhutdin A. Abdullaev, Sergey I. Glukhov, Azhub I. Gaziev

**Affiliations:** Institute of Theoretical and Experimental Biophysics, Russian Academy of Sciences, Pushchino, 142290 Moscow Region, Russia; s.glukhov@iteb.ru (S.I.G.); gaziev@iteb.ru (A.I.G.)

**Keywords:** radiation, melatonin, nDNA-repair, mtDNA-mutations, oxidation stress, protection, mitigation, H_2_O_2_, ATP, MDA, GSH

## Abstract

We used various markers to analyze damage to mouse tissues (spleen and cerebral cortex) which have different proliferative activity and sensitivity to ionizing radiation (IR). We also assessed the degree of modulation of damages that occurs when melatonin is administered to mice prior to and after their X-ray irradiation. The data from this study showed that lesions in nuclear DNA (nDNA) were repaired more actively in the spleen than in the cerebral cortex of mice irradiated and treated with melatonin (N-acetyl-5-methoxytryptamine). Mitochondrial biogenesis involving mitochondrial DNA (mtDNA) synthesis was activated in both tissues of irradiated mice. A significant proportion of the newly synthesized mtDNA molecules were mutant copies that increase oxidative stress. Melatonin reduced the number of mutant mtDNA copies and the level of H_2_O_2_ in both tissues of the irradiated mice. Melatonin promoted the restoration of ATP levels in the tissues of irradiated mice. In the mouse tissues after exposure to X-ray, the level of malondialdehyde (MDA) increased and melatonin was able to reduce it. The MDA concentration was higher in the cerebral cortex tissue than that in the spleen tissue of the mouse. In mouse tissues following irradiation, the glutathione (GSH) level was low. The spleen GSH content was more than twice as low as that in the cerebral cortex. Melatonin helped restore the GSH levels in the mouse tissues. Although the spleen and cerebral cortex tissues of mice differ in the baseline values of the analyzed markers, the radioprotective and radiomitigative potential of melatonin was observed in both tissues.

## 1. Introduction

Ionizing radiation is often used in the treatment of various tumor diseases. However, healthy tissues may also be damaged by radiation, including the induction of short-term and long-term effects and the appearance of secondary tumors [1]. Medical staff who use IR sources for diagnosis and therapy and professionals involved in the production of nuclear technologies can be exposed to radiation. A significant number of people can be exposed to IR during radiological or nuclear technology incidents or accidents. The impact of cosmic irradiation on astronauts is also a critical factor for space flights outside the Earth’s orbit [2]. Therefore, the search for and study of radioprotectors, radiomitigators, and means of treating radiation injuries remain rather topical problems. The development of such drugs has been the focus of attention of radiobiologists and radiologists for decades [3]. Antioxidant compounds account for a significant proportion of preclinical studies of radioprotectors and radiomitigators, since radiation exposure to cells is associated with the induction of prolonged intracellular oxidative stress [4]. Melatonin (N-acetyl-5-methoxytryptamine) was found to be extremely effective among the numerous compounds that passed preclinical tests as radioprotectors as it reduced the in vitro and in vivo effects of IR [5,6]. Currently, melatonin (MEL) is widely used clinically as an adaptogenic drug that normalizes circadian rhythms and is increasingly finding clinical use as an adjuvant in the radiotherapy of tumors [7,8,9]. According to the analysis of data from a large number of studies, the provisions on the possibility to use melatonin to protect astronauts from hard cosmic irradiation have been substantiated [10]. In this case, it happens that the main risks are mostly associated with the possible consequences of cosmic irradiation’s effects on the central nervous system and spleen, which lead to potential neurological disorders, degenerative effects, and decrease in the immune system and affect many aspects of the crew’s health [11,12]. As is known, that brain and spleen tissues exhibit different radiosensitivity [3]. Many years ago (in 1906) J. Bergonié and L. Tribondeau proposed a “rule” stating that ionizing radiation is more harmful to cells with a faster turnover. Therefore, there is a relationship between the radiosensitivity and proliferative activity of various tissues [13]. According to this rule, the brain can be considered a radioresistant tissue, and the spleen can be considered a radiosensitive tissue. Today, it is a generally accepted understanding [3]. We can agree with this only based on data on structural disorders and cell death in these tissues, since functional physiological disorders in the brain are observed even under the action of small doses of radiation [14]. It should also be noted that a significant amount of research is devoted to the study of the modulation of radiation damage to the brain under the action of various compounds, including MEL, while similar studies devoted to the modulation of spleen damage are rather limited.

This study is devoted to the comparative assessment on a number of markers, damages in the cerebral cortex and spleen tissues of mice after irradiation of their whole bodies with X-rays, and the modulation of these damages when MEL was administered before and after irradiation. Nuclear DNA (nDNA) and mitochondrial DNA (mtDNA) damage and repair, change in the number of mtDNA copies, H_2_O_2_, ATP, reduced glutathione (GSH) as a marker of the antioxidant system, and malondialdehyde (MDA) as a marker of oxidative stress were used as markers.

## 2. Materials and Methods

### 2.1. Chemicals

All chemicals were of the “high purity” category from the Alamed company, Moscow, Russia and from the Sigma-Aldrich company, St. Louis, MO, USA. All solutions were prepared in deionized water obtained from the Milli-Q system (Millipore, Bedford, MA, USA). Melatonin (MEL) was obtained from Sigma-Aldrich, St. Louis, MO, USA.

### 2.2. Animals and Their Irradiation

Male mice C57BL/6 at the age of 2 months weighing 20–22 g were obtained from Stolbovaya nursery (Settlement Stolbovaya; Moscow, Russia). The mice were used in experiments after 7 days of acclimatization in the animal room. All experiments with animals were performed in accordance with the European Convention for the protection of vertebrate animals used for experimental and other scientific purposes, Directive 2010/63/EU. The protocol was approved by the Committee on Biomedical Ethics of the Institute of Theoretical and Experimental Biophysics of the Russian Academy of Sciences/the Physiology Section of the Russian Committee on Bioethics (Protocol N° 20 dated 9 February 2021). The animals were fed a special diet for mice and rats and had free access to clean drinking water. The animals were irradiated at the Research Equipment Sharing Center, a group of radiation sources of the Institute of Cell Biophysics of the Russian Academy of Sciences, on a RUT-250-15-1 X-ray machine (280 kVp, 20 mA) with AL and Cu filters of 1 mm with a dose rate of 1 Gy/min. The animals were irradiated in plastic containers at a dose of 5 Gy. The irradiation of mice was carried out for 5 min.

### 2.3. Administration of Melatonin to Mice and Collection of Tissues for Analysis

A freshly prepared MEL solution was used for administration. To do this, 250 mg of MEL was dissolved in boiled drinking water (at room temperature) containing 0.1% dimethyl sulfoxide (DMSO). The final concentrations of this solution were 2.5% MEL and 0.1% DMSO. Mice were orally treated with 100 μL of this solution, corresponding to doses of MEL of 125 mg/kg and DMSO of 0.1 mg/kg of a mouse’s body weight [15]. A 0.1% DMSO solution was also prepared separately for administration to control groups of mice. The solutions were administered to groups of mice 30 min before irradiation or 20 min after irradiation. Each individual analysis group consisted of 5–6 mice. The preparation was additionally injected into drinking water (0.3 mg/mL) within 24 and 48 h for mice that were treated with MEL after irradiation, given the short clearance of MEL [15]. To isolate the cerebral cortex and spleen tissues, mice were sacrificed by decapitation 15 min and 24 and 48 h after irradiation. Groups of unirradiated and irradiated mice not treated with MEL were used as controls. The spleen and brain tissue (cortex) were separated with a scalpel on ice immediately after decapitation, then were frozen and stored at −80 °C until analysis.

### 2.4. DNA Isolation and Purification

Tissues were homogenized in a glass homogenizer and DNA was isolated using the QIAGEN Genomic-tip Kit and Genomic DNA Buffer (QIAGEN, Hilden, Germany). The amount of DNA in all cases was determined by its reaction with the PicoGreen reagent according to the manufacturer’s protocol (Molecular Probes Inc., Eugene, OR, USA) and fluorescence was registered on an NanoQuant Infinite M200 instrument (Tecan Group Ltd., Grödig/Salzburg, Austria). DNA samples for mitochondrial genome PCR-analysis were incubated within 20 min at 25 °C in TE buffer with XhoI restriction endonuclease (New England Biolabs, Ipswich, MA, USA). XhoI endonuclease initiates a break at the site of the CTCGAG hexamer of the supercoiled mtDNA outside the amplified region and leads to relaxation of the mtDNA, making the selected region available for PCR [16].

### 2.5. Analysis of Damage and Repair of Mitochondrial DNA and Nuclear DNA

To determine the damage and repair of nDNA and mtDNA, we used the long amplicon quantitative polymerase chain reaction (LA-QPCR) method [17] taking into account our previous experience [18]. In these analyses, we used (2U/μL) KAPA Long Range Hot Start Kit (KAPA Biosystems, Humboldt County, CA, USA). LA-QPCR was used to amplify a 8.7 kb region of nDNA and 10.9 kb of mtDNA. For amplification of a long fragment of mtDNA (10.9 kb), the standard thermocycler program included initial denaturation at 94 °C for 5 min, with 18 cycles of 94 °C for 30 s and 68 °C for 12.5 min, and with a final extension at 72 °C for 10 min. To amplify a long fragment of nDNA (8.7 kb), the thermocycler profile included initial denaturation at 94 °C for 5 min, and 28 cycles of 94 °C for 30 s and 68 °C for 12 min, with a final extension at 72 °C for 10 min. Preliminary assays were carried out to ensure the linearity of PCR amplification with respect to the number of cycles and DNA concentration. Since the amplification of a small region would be relatively independent of oxidative DNA damage (low probability), a small DNA fragment for nDNA (110 bp) and for mtDNA (117 bp) was also amplified for normalization of the data obtained with the large fragments, as described previously [18,19]. PCR analyses were performed in triplicate for each DNA sample. All of the amplified products were resolved and visualized using agarose gel electrophoresis and quantitated with an Image Quant (Molecular Dynamics, Waukesha, WI, USA) or VarsaDoc (Bio-Rad, Hercules, CA, USA). The data were plotted as histograms with relative amplification, such as the *y*-axis, which was calculated by comparing the values of exposed samples with the control. All primers are presented in Table 1.

### 2.6. Quantitative Analysis of Mitochondrial DNA Copies Relative to the Nuclear DNA

Quantitative analysis of mtDNA was carried out by real-time PCR with TaqMan oligonucleotides on a Prism 7500 thermal cycler (Applied Biosystems, Foster City, CA, USA) [20]. The changes in the relative quantity of mtDNA with respect to nDNA were determined as a ratio between the number of copies of the mitochondrial *ND4* gene and that of the *GAPDH* gene of nDNA in the same test tube. The 2^−ΔΔCT^ method was used for analysis. PCR tests were carried out in triplicate for each DNA sample. The following PCR program was used: 5 min at 95 °C followed by 40 cycles (95 °C for 30 s, annealing and elongation at 60 °C for 1 min). The results are presented as a percentage of data compared to unirradiated mice (taken as 100%). The PCR primers used in this study are given in Table 1.

### 2.7. Surveyor Nuclease Assay of mtDNA Mutant Copies

To evaluate the relative level of mutant copies of mtDNA isolated from brain tissue, we used the Surveyor^®^ Mutation Detection Kit (Transgenomic, Omaha, NE, USA), as described in [21,22]. To estimate mutations in mtDNA, a region including the *ND3* gene (534 bp) was chosen for amplification. The PCR primers employed in this study are given in Table 1. PCR was carried out by a programmed thermocycler Thermal Cycler 2720 (Applied Biosystems, Foster City, CA, USA). PCR was performed in a 25 μL volume containing 1.0 ng of total DNA, 75 mM of Tris-HCl, a pH of 8.8, 20 mM of (NH_4_)_2_SO_4_, 2.5 mM of MgCl_2_, 200 μM of each dNTP, 250 nM of each primer, 0.01% tween-20, and 1.0 unit of total mixture of Taq and Pfu polymerases (Thermo Scientific, Pittsburgh, PA, USA). PCR was initiated by a “hot start” after initial denaturation for 4 min at 94 °C. The amplification was carried out in 40 cycles under the following conditions: 30 s at 94 °C, 30 s at 62 °C, and 1 min at 72 °C; the final extension step of 4 min was at 72 °C. After the PCR was completed, all amplification products were diluted to an equal concentration. To obtain heteroduplex DNA, equal volumes (7 μL) of PCR products of mtDNA amplification from control and exposed mice were mixed. The mixtures were heated at 95 °C for 10 min and cooled slowly to 40 °C for 70 min at a rate of 0.3 °C/min. Then, 1/10 volume of 0.15 M MgCl_2_ solution, 1 μL of Surveyor Enhancer S, and 1 μL of Surveyor Nuclease S were added to the heteroduplex mixture. The mixture was incubated at 42 °C for 60 min. The reaction was stopped by adding 1/10 volume of stop solution. Nuclease digestion products were analyzed by electrophoresis in a 2.0% agarose gel stained with ethidium bromide. PCR tests of heteroduplexes were carried out in triplicate for each DNA sample. The fluorescence intensity of DNA bands in the gels was registered by the AlphaImager Mini System (Alpha Innotech, Santa Clara, CA, USA). The ratio of the cleavage products’ fluorescence to the total intensity of fluorescence of DNA bands in the gel (% of Surveyor Nuclease cleaved DNA) was calculated using the ImageJ software package (Wayne Rasband, Kensington, MD, USA).

### 2.8. Determination of Hydrogen Peroxide Level

A Fluorimetric Hydrogen Peroxide Assay Kit 165 (Sigma-Aldrich Co., St. Louis, MO, USA) was used for the quantitative measurement of hydrogen peroxide (H_2_O_2_) in mice tissues. This kit uses peroxidase substrate that generates a red fluorescent product that can be analyzed in 96-well black transparent bottom microplates. All analyses were performed in accordance with the recommendations of the manufacturer. The amount of H_2_O_2_ was calculated on the basis of a standard curve obtained using a concentration range of an H_2_O_2_ solution obtained by diluting a 30% H_2_O_2_ solution with ultrapure water. Each test sample was run in triplicate. Data were obtained from 6 mice in each group. The amount of H_2_O_2_ was expressed in nmol per mg of protein using a standard curve. Protein was assessed in these and other analyses by the method of Lowry et al. [23] using bovine serum albumin as a standard.

### 2.9. ATP Analysis

The ATP content was determined following the recommendations indicated in [24]. ATP was extracted from tissue homogenates after the removal of proteins with TE buffer saturated with phenol. ATP level was measured using a luciferin–luciferase kit with a GloMax 96 Microplate Luminometer (Promega, E6521, Madison, WI, USA). ATP concentration was assessed using a standard curve in nmol per mg of protein. Data were normalized to total protein, and tissue ATP levels were expressed in μmol per 100 mg of protein.

### 2.10. Determination of Lipid Peroxidation

The lipid peroxidation level was judged by changes in malondialdehyde (MDA) content after reaction with thiobarbituric acid (TBA) by the method of Buget and Aoust [25]. For this purpose, the cerebral cortex and spleen tissues of mice were homogenized in lysis buffer (50 mM Tris-Cl, 1% NP-40, 0.2% sodium deoxycholate, 0.1% SDS, 150 mM NaCl, and 1 mM EDTA). Then, one volume of tissue lysate was mixed with two volumes of TBA reagent (15% TCA, 0.375% TBA, and 0.25 N HCl), followed by incubation at 90 °C for 30 min. After cooling, the reaction mixture was centrifuged at 10,000 rpm for 15 min. The supernatant absorbance was measured at 533 nm with respect to the blank. The amount of lipid peroxidation was calculated from the MDA level in nmol per milligram of protein (nmol/mg of protein).

### 2.11. Determination of Glutathione Level (GSH)

Tissues were homogenized in lysis buffer (50 mM Tris-Cl, 1% NP-40, 0.2% sodium deoxycholate, 0.1% SDS, 150 mM NaCl, and 1 mM EDTA) as indicated in the determination of lipid oxidation [25]. A total of 1.8 mL of 0.05 M EDTA and 3 mL of a precipitator (containing 1.67 g of HPO_3_, 0.2 g of disodium EDTA salt, and 30 g of NaCl per liter of water) were added to 0.2 mL of tissue homogenate. After thorough mixing, the solution was kept for 5–7 min and then centrifuged. This step promotes the separation of GSH (in the supernatant) from the rest of the proteins and other cellular elements (in the sediment). Then, two volumes of 0.3 M Na_2_HPO_4_ solution and 0.5 volumes of 4 mM DTNB (5,5′-dithiobis-2-nitrobenzoic acid) were added to one volume of the supernatant [26]. Absorbance was determined at 412 nm against a mixture of solutions without biomaterial additives (blank). GSH was expressed in nmol per mg of protein using a standard curve.

### 2.12. Statistical Analysis

All numerical results are expressed as the mean ± SEM of 5–6 independent experiments and *p* < 0.05 was considered statistically significant. The statistical analyses were performed using GraphPad Prism 8.0 software (San Diego, CA, USA).

## 3. Results

### 3.1. Damage and Repair of Nuclear DNA and Mitochondrial DNA following Irradiation

**1.** As established in a number of studies, exogenous melatonin is a powerful antioxidant and has *in vitro* and *in vivo* radioprotective and radiomitigator effects [5,6]. Melatonin also exhibits a wide range of antioxidant defense reactions at various cellular levels. It helps to reduce oxidative stress caused by active forms of oxygen and nitrogen (RONS) and acts as an absorber of free radicals [27,28]. Therefore, it is of interest to elucidate changes in the most important markers of radiation damage in tissues with different radiosensitivity and proliferative activity in animals when they are administered with melatonin. In our study on mice, the spleen and cerebral cortex were taken as such tissues. In this study, mice were irradiated on a RUT-250-15-1 X-ray machine (280 kVp, 20 mA) with AL and Cu filters of 1 mm with a dose rate of 1 Gy/min. As is known, the most important marker of radiation exposure to living organisms is DNA damage. To determine nDNA and mtDNA damage, we used the method of quantitative PCR with a long amplicon (LA-QPCR) [17]. The presence of damage such as modified bases, single-strand and double-strand breaks, or DNA–protein crosslinking can block the activity of KAPA Biosystems’ DNA polymerase. Thus, this method allowed us to assess the overall level of DNA damage.

**Figure 1:**
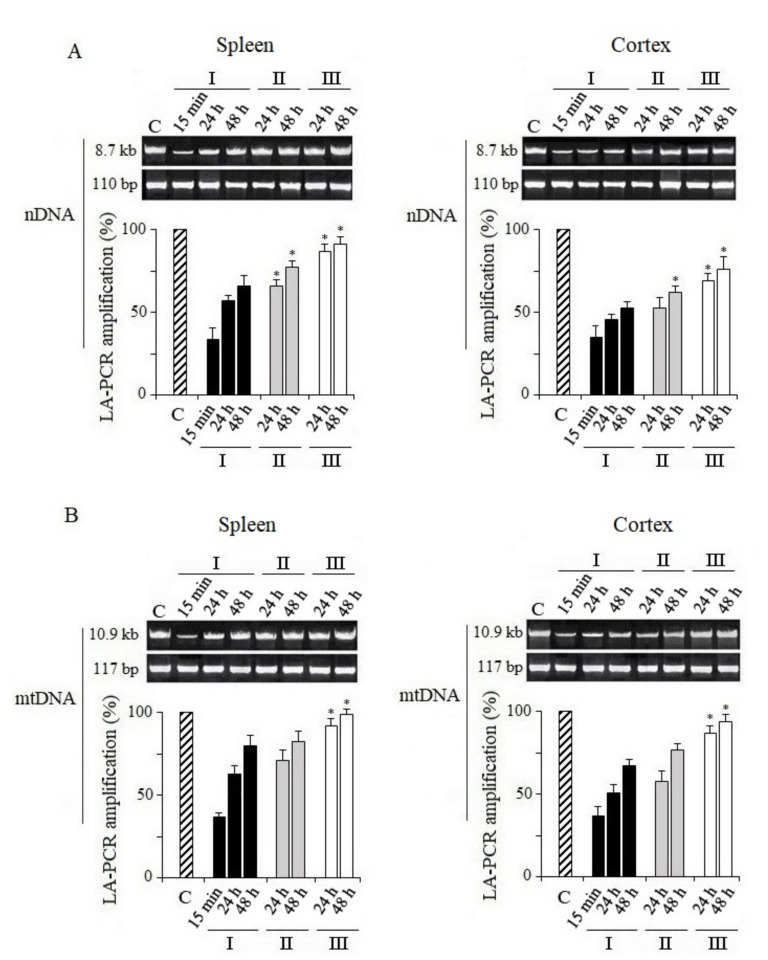
Analysis of damage and repair of nuclear DNA and recovery of mitochondrial DNA. Long fragments of nDNA (8.7 kb) and mtDNA (10.9 kb) were measured. These data were normalized by the measured levels of the short fragment of nDNA (110 bp) and mtDNA (117 bp), obtained using the same DNA sample. (**A**) Quantitative analysis of the LA-QPCR amplicons of nDNA extracted from spleen and cerebral cortex. (**B**) Quantitative analysis of the LA-QPCR amplicons of mtDNA extracted from spleen and cerebral cortex. Data are presented in % to control (C). Here and in other figures: the dose of X-ray irradiation of mice was 5 Gy and MEL was administered to mice before and after irradiation as a single dose of 125 mg/kg. Electropherogram samples of synthesized amplicons are presented above the histograms. The numbers (15 min, 24 h, 48 h) above and below indicate the time after irradiation. I—mice without MEL administration; II—MEL administration before irradiation; III—MEL administration after irradiation. The data are presented as mean ± SEM of 5–6 independent experiments. Statistical significance was set at * *p* < 0.05.

**2.** The amplification products of long sections of nDNA and mtDNA from the tissues of unirradiated mice were taken as 100% control. It can be seen that the level of synthesized products of nDNA and mtDNA LA-QPCR from the mice’s spleens and cerebral cortexes 15 min after irradiation was significantly lower than that of the unirradiated mice (Figure 1). Such a reduction in LA-QPCR products indicates that these amplifiable areas of nDNA and mtDNA contained damages capable of blocking KAPA Long Range Rapid PCR DNA polymerase (KAPA Biosystems, Wilmington, MA, USA). The preservation of low levels of amplification of nDNA and mtDNA regions indicates the presence of non-repaired damages in them. However, there was an increase in LA-QPCR products by 24 and 48 h post-radiation time, which indicates the functioning of DNA damage repair processes. According to the results obtained, nDNA and mtDNA from the tissues of mice treated with MEL before IR-radiation and after irradiation had significantly less damages capable of blocking KAPA Long Range DNA polymerase. As can be expected, this shows that MEL contributes to the DNA damage reduction (Figure 1). nDNA and mtDNA from the tissues of mice treated with MEL before irradiation and after irradiation had significantly less damages capable of blocking KAPA Long Range DNA polymerase. As can be expected, this also shows that MEL contributes to the DNA damage reduction. The results obtained show that the nDNA repair occurs more actively in the spleen and cerebral cortex tissues of mice treated with MEL after irradiation. When comparing the LA-QPCR amplification data of nDNA from two tissues of mice irradiated and treated with MEL, it seems that the nDNA repair in the spleen tissue for the indicated periods of post-radiation time was more active than in the cerebral cortex (Figure 1).

**3.** According to the experiment results, we can also conclude that mtDNA in the spleen and the cerebral cortex was actively restored, especially in mice that were treated with MEL after irradiation (Figure 1). However, if the increase in the synthesis of the LA-QPCR product of nDNA during the post-irradiation period was due to the repair of nDNA damages that inhibited KAPA Long Range DNA polymerase, this is unlikely to be the reason for the sharp increase in the synthesis of LA-QPCR products of mtDNA from the same tissues of mice. It is known that only base excision repair (BER) effectively functions in mammalian mitochondria [29]. Other pathway of repairing mutagenic mtDNA damage do not function in mammalian mitochondria. Moreover, double-strand breaks (DSBs) of mtDNA in mammalian cells are not repaired [30,31], and damaged mtDNA can undergo degradation [32]. In this experiment, we most likely registered the activation of mitochondrial biogenesis with the mtDNA synthesis (Figure 1). To test this assumption, we decided to continue experiments to elucidate the effect of MEL on the quantitative content of mtDNA relative to nDNA in the spleen and cerebral cortex tissues of mice exposed to radiation.

### 3.2. Effect of Melatonin on Mitochondrial Biogenesis in Tissues of X-Irradiated Mice

**Figure 2:**
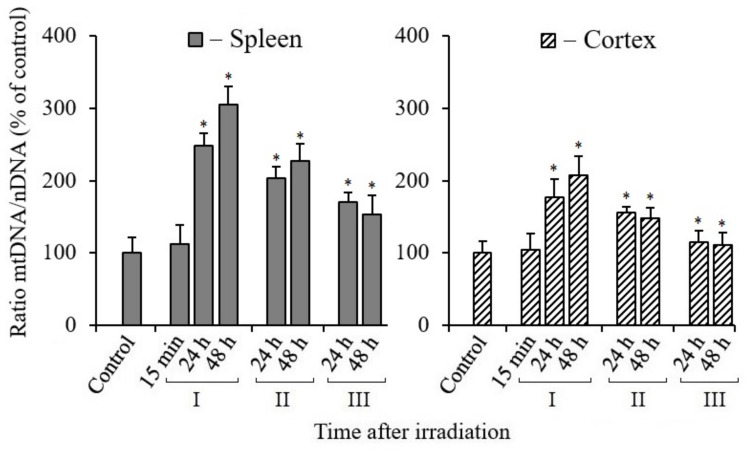
Ratio of mtDNA/nDNA in the tissues of the spleen and cerebral cortex of mice after their irradiation. The *y*-axis shows the percentage (%) of the change in mtDNA to nDNA ratio relative to control. The numbers (15 min, 24 h, 48 h) on *X*-axis indicate the time after irradiation. I—mice without MEL administration; II—MEL administration before irradiation; III—MEL administration after irradiation. The data are presented as mean ± SEM of 5–6 independent experiments. Statistical significance was set at * *p* < 0.05.

A change in the number of mtDNA copies or the ratio of mtDNA/nDNA is the most important criterion for assessing mitochondrial biogenesis in tissues or cells [30,31]. The results of the analyses obtained by the real-time PCR method show that the number of mtDNA copies increased in the spleen and cerebral cortex tissues of mice 24 and 48 h after their irradiation with a dose of 5 Gy in comparison with the data of the control (non-irradiated) animals group (Figure 2). As judged from the number of mtDNA copies, the enhancement of mtDNA synthesis was more pronounced in the spleen tissue than in the cerebral cortex of the irradiated mice. It should also be noted that the content of mtDNA in the tissues of mice, for a 15-min time after irradiation, remained at the level of the data from the control non-irradiated mice. These results indicate that mtDNA synthesis and, accordingly, mitochondrial biogenesis were activated much later; we registered their increase 24 and 48 h after irradiation. At the same time, we can see that when MEL was administered, the synthesis of mtDNA molecules occurred less actively than in the data obtained in irradiated mice without the administration of MEL. This gives the impression that MEL partially suppresses IR-induced mtDNA synthesis in the tissues of the spleen and cerebral cortex. In fact, most likely, this is the result of a decrease in the level of RONS generated by dysfunctional mitochondria under the influence of MEL. At the same time, the inhibition effect of IR-induced mtDNA synthesis upon administration of MEL to animals after irradiation was more pronounced in comparison with the data of the group of mice treated with MEL before irradiation. Based on the data obtained, it can be assumed that upon initiation of replicative synthesis involving a damaged mtDNA template and with the participation of DNA polymerase γ and DNA polymerase θ in mitochondria [32,33], the appearance of new copies of mtDNA with mutations and deletions in the tissues of mice after irradiation with IR can be expected.

### 3.3. Analysis of Mitochondrial DNA Mutant Copies

**Figure 3:**
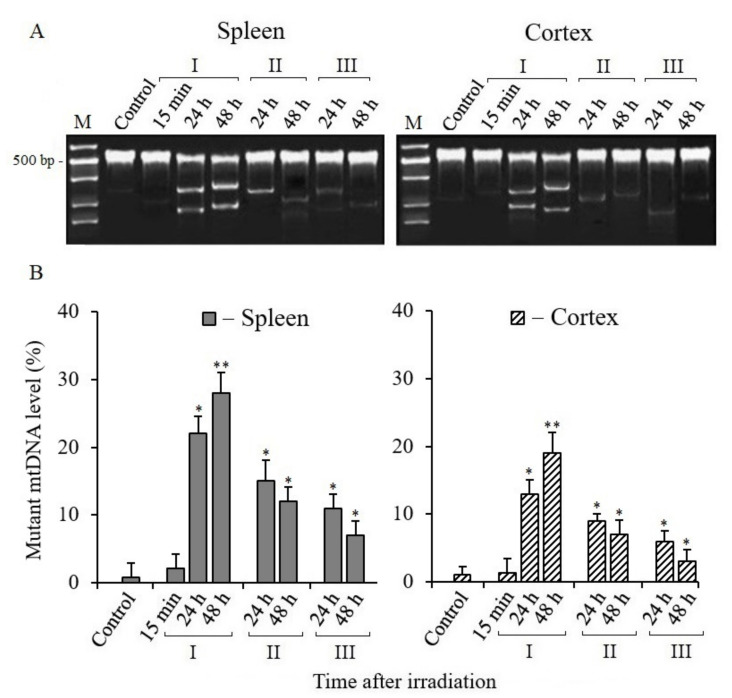
Detection of mtDNA mutant copies of spleen and cerebral cortex tissues in mice 15 min, 24, and 48 h after X-ray irradiation. (**A**) Electrophoresis of cleavage products obtained by Surveyor nuclease digestion of heteroduplexes of mtDNA PCR amplicons from spleen and cerebral cortex tissues. (**B**) Percentage of Surveyor nuclease cleaved heteroduplexes of PCR amplicons of mtDNA (ND3 gene, 534 bp). I—mice without MEL administration; II—MEL administration before irradiation; III—MEL administration after irradiation. The data are presented as mean ± SEM of 5–6 independent experiments. Statistical significance was set at * *p* < 0.05, ** *p* < 0.01.

As noted above, with the exception of BER, other DNA repair pathways are not involved in repairing mtDNA damage in mammalian cells [26]. Therefore, the observed increase in the number of mtDNA copies in the tissues of irradiated mice (Figure 2) suggested that it was associated with increased mtDNA mutagenesis. Our subsequent analyses confirmed this assumption. Electropherograms of the Surveyor nuclease digestion products of mtDNA PCR amplicon heteroduplexes and their quantitative analysis are shown in Figure 3. The quantitative analysis of the cleavage products of heteroduplexes showed that the level of mtDNA mutant copies significantly increased in the spleen and cerebral cortex tissues of mice within 24–48 h after irradiation (Figure 3B). The number of mutant copies in the spleen tissue increased to 30% by 48 h post-radiation time, and it also increased to 20% in the cerebral cortex tissue relative to the control. On the other hand, the data from the analysis of the mtDNA mutant copies number in the tissues of mice treated with MEL before and after irradiation were significantly lower than those from mice that were not treated with MEL. It should also be noted that a significant decrease in the mtDNA mutant copies number was recorded, as can be seen, in the cerebral cortex tissue when MEL was administered into mice after irradiation in comparison with data from the spleens of groups of irradiated mice that were treated with MEL.

### 3.4. Changes in H_2_O_2_ Content in Tissues of X-Irradiated Mice

**Figure 4:**
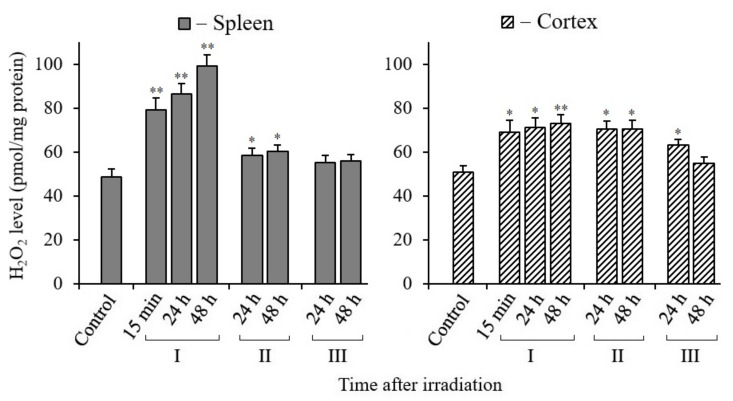
Changes in the H_2_O_2_ content in spleen and cerebral cortex tissues of mice 15 min, 24, and 48 h after their exposure to X-rays. I—mice groups without MEL administration; II—MEL administration before irradiation; III—MEL administration after irradiation. The data are presented as mean ± SEM of 5–6 independent experiments. Statistical significance was set at * *p* < 0.05; ** *p* < 0.01.

As is known, mitochondria and a number of extramitochondrial oxidases generate various reactive oxygen and nitrogen species (RONS). However, not all RONS can diffuse through the membranes of mitochondria or other organelles and reach the cell nucleus, since most of them migrate only over short distances. H_2_O_2_ molecules are the most stable and capable of migrating over long distances (1 μm or more) [34,35]. Therefore, we decided to determine changes in oxidative stress in the spleen and cerebral cortex tissues of irradiated mice by the level of hydrogen peroxide. The analysis results are shown in Figure 4. The data show that H_2_O_2_ production increased more sharply in the spleen tissue of mice during 24–48 h of the post-radiation period. At the same time, with the introduction of MEL, the level of H_2_O_2_ in the spleen significantly decreased. In the tissue of the cerebral cortex, the tendency for changes in the content of H_2_O_2_ is the same as in the spleen, but less pronounced. Here (Figure 4) it can be seen that, after the irradiation of mice, the H_2_O_2_ level increased immediately after 15 min and this level remained for 24 and 48 h. At the same time, we observed a decrease in the H_2_O_2_ level after only 48 h in the cerebral cortex tissue of mice treated with MEL after irradiation.

### 3.5. Changes in ATP Content in Tissues of X-Irradiated Mice

**Figure 5:**
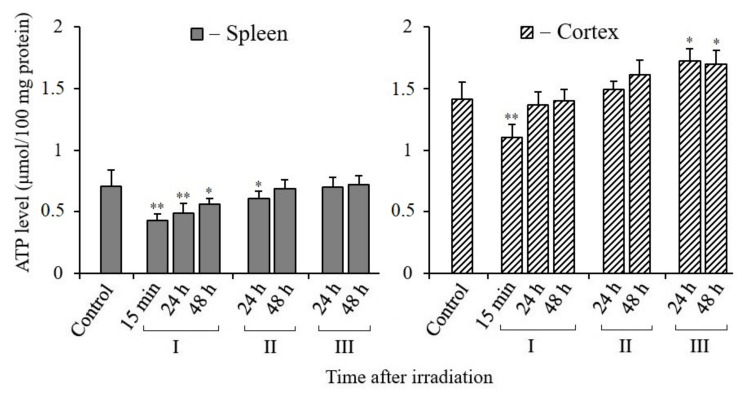
Changes in the ATP content in spleen and cerebral cortex tissues of mice 15 min, 24, and 48 h after their irradiation. I—mice groups without MEL administration; II—MEL administration before irradiation; III—MEL administration after irradiation. The data are presented as mean ± SEM of 5–6 independent experiments. Statistical significance was set at * *p* < 0.05; ** *p* < 0.01.

Maximum energy support is required for DNA repair and cell recovery. This can ensure the synthesis of ATP in functionally active mitochondria [36]. Therefore, it is very important to evaluate the change in the ATP content in the tissues of irradiated mice and the effect of MEL on the correction of its synthesis level. The results of our analyses show that the content of ATP in the spleen tissue was approximately two times less per unit mass of tissue compared to its content in the cerebral cortex tissue of control and irradiated mice (Figure 5). The observed difference was obviously due to the unequal content of mitochondria in these tissues. Nevertheless, the post-radiation changes in the ATP content in both tissues were relatively similar. It can be seen that the ATP content in both tissues sharply decreased in the initial period after irradiation, especially after 15 min. However, a tendency towards restoration of the ATP content in both tissues of the irradiated mice was observed after 24 and 48 h of post-radiation time. Moreover, the restoration of the ATP content in the tissues of the mice that were treated with MEL before and after irradiation was more active. This is best seen in the results obtained on the cerebral cortex tissues. Thus, we can conclude that MEL contributes to the maintenance of mitochondrial functions and the synthesis of the required level of ATP in the spleen and cerebral cortex tissues of irradiated mice.

### 3.6. Changes in MDA Content in Tissues of X-Irradiated Mice

In radiation biology, an increase in the level of the lipid oxidation product malondialdehyde (MDA) in cells or tissues is considered as one of the most important markers of radiation damage. This marker indicates the occurrence of oxidative stress.

**Figure 6:**
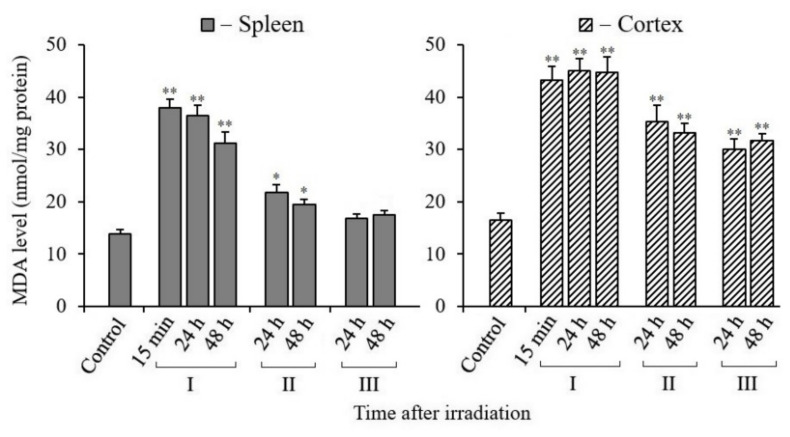
Changes in the MDA content in spleen and cerebral cortex tissues of mice 15 min, 24, and 48 h after their exposure to X-rays. I—mice without MEL administration; II—MEL administration before irradiation; III—MEL administration after irradiation. The data are presented as mean ± SEM of 5–6 independent experiments. Statistical significance was set at * *p* < 0.05, ** *p* < 0.01.

The results of our analyses gave quite different results of the content of MDA in the tissues of the spleen and cerebral cortex of mice exposed to X-rays (Figure 6).

Administration of MEL to mice before and after irradiation promoted a significant decrease in MDA in the spleen tissue. Similar results were obtained by MDA analyses in the cerebral cortex tissue of the same mice. However, the results of the brain tissue analyses were quantitatively different from those of the spleen tissue analyses. First of all, the MDA level in the cerebral cortex tissue was higher in comparison with the data of the spleen analyses. Moreover, the administration of MEL to mice before and after irradiation in the brain tissue retained an increased content of MDA, although it was significantly lower than that in the analysis data from the tissues of mice that were not treated with MEL. It can also be noted that the data obtained from MDA analyses both in the spleen tissue and in the brain tissue of irradiated mice that were treated with MEL after irradiation were lower than the results obtained in the tissues of mice that were treated with MEL before irradiation.

### 3.7. Changes in Glutathione Content in Tissues of X-Irradiated Mice

**Figure 7:**
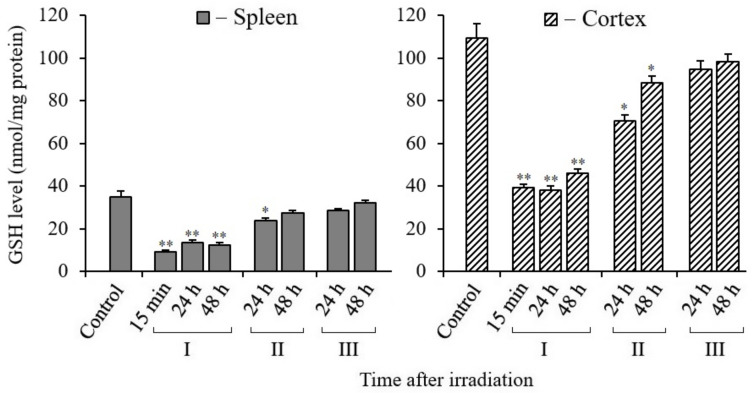
Changes in the GSH in spleen and cerebral cortex tissues of mice 15 min, 24, and 48 h after their irradiation. I—mice without MEL administration; II—MEL administration before irradiation; III—MEL administration after irradiation. The data are presented as mean ± SEM of 5–6 independent experiments. Statistical significance was set at * *p* < 0.05, ** *p* < 0.01.

Reduced glutathione (GSH) is an essential non-enzymatic antioxidant that plays a prominent part in determining cell radiosensitivity. A decrease in the content of GSH in tissues or in the blood is considered as a marker of a decrease in the level of antioxidants in the body as a result of radiation exposure. The results of our analyses show that there was a sharp decrease in glutathione in the spleen and cerebral cortex tissues of mice after irradiation of the whole body with X-rays (Figure 7). These data also show that the content of GSH in the spleen was more than two times less than that in the cerebral cortex tissue. At the same time, reduced levels of GSH were retained in both tissues during the post-radiation time (up to 48 h). We observed an active increase in the content of reduced GSH in the tissues of these mice only after oral administration of MEL to mice before or after irradiation. At the same time, the results show that the restoration of GSH level in the cerebral cortex tissue occurred more actively in mice treated with MEL after irradiation.

## 4. Discussion

In a normally functioning cell, DNA is constantly subject to oxidation and “spontaneous” hydrolytic degradation [37]. RONS cause a lot of damage in DNA, including base modifications, the destruction of deoxyribose, formation of apurinic/apyrimidinic sites and single-strand breaks (SSBs) [38]. In addition, double-strand breaks (DSBs) can also form in DNA as a result of a close match of SSBs or in the process of repair of closely spaced damaged bases on complementary strands of a double helix [39]. When IR is exposed to cells, DNA damage is induced much more (depending on the dose of IR). Moreover, there is a sharp increase in the production of RONS in irradiated cells, which can last from several minutes to tens of days, depending on the radiation dose [40]. Therefore, supporting the activity of DNA repair systems and the level of antioxidants play a crucial role in the fate of the irradiated organism. It is obvious that in the regulation of these processes, along with other protective systems of the cell, melatonin can play a primary role [41].

The review article by Galano et al. [41] analyzes the data of many studies on the role of MEL in protecting DNA from oxidative damage. It is shown here that MEL provides cleaning of free radicals and other forms of RONS from cells and activates enzymes involved in the BER. MEL activates the expression of genes encoding DNA repair enzymes and antioxidant enzymes, but suppresses the activity of pro oxidant enzymes. Thus, it is clear that MEL provides protection of the nuclear genome in different directions [41].

It has recently been reported that MEL not only protects DNA, to a large extent, from mutagenic damage, but also from the induction of DNA DSBs, which are lethal events for the cell if they are not repaired. Thus, in patients undergoing computed tomography (CT), DNA DSBs induction was recorded in blood lymphocytes. Moreover, in the group who received a single oral dose of 100 mg of MEL 5–10 min before and 30 min after CT examination, these DNA damages was not recorded [42]. These results are confirmed in another study. The authors observed DNA DSBs in lymphocytes when exposed to IR at doses of 10 mGy and 100 mGy. Administration of 100 mg of MEL to patients before irradiation caused a decrease in DNA DSBs levels [43]. In another study, when incubating human blood lymphocytes in an environment with the addition of radioactive iodine I^131^ for 2 h in the presence of MEL, the number of induced DNA DSBs decreased by 40% relative to the control (lymphocytes incubated with I^131^ without MEL) [44].

As described above, for a comparative assessment of damage and repair of nDNA and mtDNA in different tissues, we used the method of quantitative PCR with a long amplicon (LA-QPCR) [17]. The presence of damage such as modified bases, SSBs, DSBs, or DNA–protein crosslinking can block the activity of KAPA Biosystems DNA polymerase and, accordingly, reduce the PCR synthesis product. The results of our analyses indicate that the repair of nDNA total damage capable of blocking KAPA Long Range DNA polymerase in the spleen and cerebral cortex of irradiated mice proceeds rather slowly within 48 h after total body irradiation, and occurs more slowly in the brain cells (Figure 1). It is known that in postmitotic cells, different DNA repair pathways are less active than in dividing cells [45]. Recently, it was shown that after irradiation of the rat head with X-rays, DSBs nDNA in the cortical neurons persisted for a long post-radiation time [46]. We also recently reported that DNA damage repair in irradiated rats is slower in cortical tissue than in hippocampal tissue [18].

The spleen is an organ of the reticuloendothelial system with proliferative activity [47]. The nDNA damage repair in the spleen tissue is more active, although it could not be completed by 48 h without the administration of MEL.

It is possible that the observed slow DNA repair within 24–48 h in the tissues of irradiated mice without the introduction of MEL was due to the occurrence of additional damage in the same DNA. These additional damages may occur as a result of the action of RONS, generated in the dysfunctional mitochondria of the same cells. With the introduction of MEL, obviously, there is a significant cleaning of these RONS. Exposure to ionizing radiation can not only cause acute radiation syndrome, but also increase the risk of developing long-term consequences. IR stimulates RONS production by mitochondria for a few hours to a few days after irradiation. This prolonged RONS generation in mitochondria can induce additional damage to nDNA and mtDNA cells after radiation exposure [40]. It has long been established that the cause of increased oxidative stress in the cells of irradiated mammals is mitochondrial dysfunction [48]. At the same time, the antioxidant activity in the tissues and blood of irradiated rodents sharply decreases [49,50].

We found increased mtDNA synthesis in mouse tissues after irradiation, clearly associated with mitochondrial biogenesis. This well-known phenomenon is the induction of biogenesis with the synthesis of mtDNA under radiation exposure to the cells of animals [51,52,53]. It is caused by the occurrence of mitochondrial dysfunction, increased oxidative stress, and a decrease in ATP synthesis, along with the emergence of increased energy needs in damaged cells.

As noted above, the processes of mtDNA damage repair occur with low efficiency in mammalian mitochondria. In these organelles, only the pathway of the BER functions efficiently [29]. The results of our analyses (Figure 2) showed an increased mtDNA synthesis in the spleen and cerebral cortex cells by 24–48 h after irradiation. As might be expected, the activity of mtDNA synthesis decreased in both tissues when mice were treated with MEL before and after irradiation, which lowered the RONS content. Various mammalian tissue cells may exhibit tissue-specific features in the activation of mitochondrial biogenesis and mtDNA synthesis, associated with their activity in the generation of ATP and RONS [54].

The subsequent results of our studies showed that in the post-radiation mitochondrial biogenesis, some of the synthesized mtDNA molecules were mutant copies. Obviously, mutations in the newly synthesized mtDNA molecules appeared during the replication of damaged mtDNA matrixes with the participation of DNA polymerase γ and DNA polymerase θ in mitochondria [35,36]. The increased levels of mtDNA mutant copies were observed in the spleen and cerebral cortex tissues of irradiated mice; however, their number significantly decreased upon the administration of MEL before and after irradiation (Figure 3). Therefore, it can be assumed that the antimutagenic effect of MEL is due to both the interception of initial RONS (the administration of MEL before irradiation) and the neutralization of RONS generated in the cells of irradiated mice (the administration of MEL after irradiation) [55]. These results are consistent with the previously obtained data of Tan et al. [56], who concluded that MEL protects mitochondria, has a regulatory effect on mitochondrial biogenesis and dynamics, and contributes to the preservation of the functions of these organelles. The increased level of mutant mtDNA copies in mammalian tissues after irradiation is due to both the low efficiency of the mtDNA repair systems and the effect of RONS; their production in the mitochondria of mammalian cells can continue over a long post-radiation period [40].

The results of a number of studies show that the mitochondrial dysfunction detected in human and animal cells after irradiation is largely associated with the induction of mutations in mtDNA genes encoding proteins of electron transport chain complexes, which continue to operate with the overproduction of RONS [57,58]. Obviously, the expression of mtDNA mutation genes leads to the synthesis of aberrant proteins. The latter can lead to perturbation of the oxidative phosphorylation system in mitochondria, with prolongs the increased generation of ROSNS and increased oxidative stress. This causes even more damage to macromolecules in the organelles and the entire cell, including nDNA. A “vicious cycle” is formed for a long period. This cycle operates in the various mammalian tissue cells at different rates and leads to the differential accumulation of mutant mtDNA copies, which, in turn, increase oxidative stress for a long post-radiation period. Thus, it can be assumed that, when IR is exposed to mammalian tissues, mitochondria containing mtDNA mutant copies become dysfunctional with enhanced RONS generation, which supports the induction of additional nDNA damage and genome instability of surviving cells, the development of degenerative diseases, aging, and oncogenesis for a long post-radiation period [59]. The mitochondrial respiratory chain is considered to be the most important cellular source providing most of the RONS in the cells of an aerobic organism [60]. However, there are other sources of RONS in mammalian cells that can be activated by radiation exposure. These include peroxisomes and many oxidases [61]. NADPH oxidases, a family of NOX enzymes that are located in various cellular compartments, can make a significant contribution to the enhancement of oxidative stress [62]. NADPH oxidases catalyze the one-electron reduction of O_2_ to produce a superoxide anion (O_2_^•−^) followed by the formation of H_2_O_2_ and hydroxyl radicals (OH^•^) [62].

Although all types of RONS are generated in irradiated cells, the greatest contribution to nDNA damage and other macromolecules is made by H_2_O_2_, OH^•^, and ONOO, which can diffuse over long distances. Especially H_2_O_2_ molecules capable of diffusing over distances are attainable by nDNA [63]. It has been noted that in physiological conditions, the level of H_2_O_2_ can reach 1–10 nM, whereas at “supraphysiological” concentrations, its content will be higher (>100 nM) [62].

In our analyses, the increase in the H_2_O_2_ level in the spleen may have been due to the low level of antioxidants compared to the cerebral cortex tissue (Figure 4). It was also reported that with an increase in the frequency of mtDNA mutations, the level of RONS may raise in the spleen [64]. Due to the specificity of this tissue, it can be noted that iron ions are released in the spleen after irradiation, which can increase the level of RONS with the induction of cell ferroptosis [65]. However, the increased H_2_O_2_ level in the spleen tissue of irradiated mice can be significantly reduced by the administration of MEL before and after irradiation. It is possible to observe not only an increase in H_2_O_2_, but also a decrease in ATP synthesis with a loss of mitochondrial membrane potential (ΔΨm) in the initial period in the cells’ mitochondria after IR exposure [61]. The ATP content decreases unevenly in different tissues of mice. We found that the decrease in ATP was more actively manifested in the spleen (by 80%) than in the brain tissue (by 20%) (Figure 5) [60]. First of all, the reason for this is that the number of mitochondria in the brain tissue of two-month-old mice is three times greater than in the spleen [62]. The content of ATP in the spleen tissue decreases after irradiation of the mice, however, unlike the cerebral cortex, with the introduction of MEL it increases only to the control level. Perhaps this is a manifestation of the tissue specificity of the mitochondrial reaction [63]. After a short-term decrease in the content of ATP in the tissues of irradiated mice in the post-radiation period, its synthesis is restored. MEL has an active effect on the restoration of ATP synthesis, as well as on the biogenesis of mitochondria in the tissues of irradiated mice.

In radiation biology, the levels of MDA (a marker of oxidative stress) and antioxidant enzymes or reduced GSH (a marker of the antioxidant system) are often used to assess changes in the redox status of cells after irradiation. There are a number of publications in the literature which show that the radioprotective effect of MEL, observed in experiments on animals, is associated with the decreased MDA and increased GSH levels in their tissues [66,67,68]. According to the results of our study, the MDA levels in the spleen and cerebral cortex tissues of mice 48 h after irradiation remain elevated compared to the non-irradiated control tissues (Figure 6). As might be expected, the data of the GSH content analyses show decreased values. The GSH level in the spleen of the control mice was much lower than in the cerebral cortex tissue (Figure 7). There is a tendency to restore the MDA and GSH content to their reference values after the administration of MEL to mice before and after irradiation. There is still an increased MDA level in the cerebral cortex tissue after the administration of MEL, although the GSH level increases more noticeably to the reference values. The high MDA level in the cerebral cortex tissue might be due to the increased content of lipids in this tissue.

## 5. Conclusions

Numerous studies show that MEL is a strong antioxidant that exhibits radioprotective and radiomitigative effects. The results of our study on the evaluation of the effect of MEL on tissues with different proliferative activity and radiosensitivity in mice exposed to IR confirm this position. The results showed that, although the tissues of the spleen and cerebral cortex of mice differ in the initial control values of the analyzed markers, the potential of radiation protection of MEL is successfully implemented in both tissues.

It should also be noted that the issue of the expediency of splenectomy in radiotherapy of tumors of intra-abdominal organs or in astronauts during long-term space flights outside the protection of the Earth’s magnetosphere is currently being discussed. Of course, the data obtained by exposure to X-rays on the body is difficult to completely extrapolate to damage to normal tissues during hadron therapy of tumors or to the effects of cosmic radiation on astronauts. Nevertheless, since oxidative stresses of different levels occur when cells are exposed to different IR (^56^Fe, protons, and X-rays) [69], it seems possible to suppress them by MEL and refrain from splenectomy.

## Figures and Tables

**Table 1:** Primers and probes used in the current study.

Locus	Primer, Probes	Accession Number	5′→3′ Sequence	Size
mtDNA		NC_005089.1	Primers for LA-QPCR	
	for		GCCAGCCTGACCCATAGCCATAATAT	
	rev		GAGAGATTTTATGGGTGTAATGCGG	10.9 kb
nDNA	for	NC_000073.7 X14061.1	TTGAGACTGTGATTGGCAATGCCT	
	rev		CCTTTAATGCCCATCCCGGACT	8.7 kb
mtDNA	for	NC_005089.1	CCCAGCTACTACCATCATTCAAGT	
	rev		GATGGTTTGGGAGATTGGTTGATGT	117 bp
nDNA	for	NC_000071.7 NM_007393.5	CTGCCTGACGGCCAGG	
	rev		GGAAAAGAGCCTCAGGGCAT	110 bp
ND4		NC_005089.1	Primers for quantitative analysis of mtDNA/nDNA	
	for		ATTATTATTACCCGATGAGGGAACC	
	rev		ATTAAGATGAGGGCAATTAGCAGT	
	probe		FAM-ACGCCTAAACGCAGGGATTTATTTCCTA-BHQ1	115 bp
GAPDH	for	NC_000072.7 NM_001289726.1	GTGAGGGAGATGCTCAGTGT	
	rev		CTGGCATTGCTCTCAATGAC	
	probe		ROX-TAAGAAACCCTGGACCACCCACCCC-BHQ2	214 bp
ND3		NC_005089.1	Primers for mtDNA mutant copies	
	for		AGCTCTCCATTTATTGATGAGG	
	rev		GAGGTTGAAGAAGGTAGATGGC	534 bp

## Data Availability

Data is contained within the article.
